# Telomerase reverse transcriptase haploinsufficiency and telomere length in individuals with 5p– syndrome

**DOI:** 10.1111/j.1474-9726.2007.00324.x

**Published:** 2007-10-01

**Authors:** Hong-Yan Du, Rachel Idol, Sara Robledo, Jennifer Ivanovich, Ping An, Arturo Londono-Vallejo, David B Wilson, Philip J Mason, Monica Bessler

**Affiliations:** 1 Departments of Internal Medicine, Washington University School of Medicine 660 S Euclid Avenue, St. Louis, MO 63110, USA; 2 Departments of Surgery, Washington University School of Medicine 660 S Euclid Avenue, St. Louis, MO 63110, USA; 3 Departments of Genetics, Washington University School of Medicine 660 S Euclid Avenue, St. Louis, MO 63110, USA; 5 Departments of Pediatrics, Washington University School of Medicine 660 S Euclid Avenue, St. Louis, MO 63110, USA; 4 Telomeres and Cancer Lab, UMR7147, CNRS-I, Curie-UPMC Paris, France

**Keywords:** 5p– syndrome, dyskeratosis congenita, haploinsufficiency, telomerase reverse transcriptase, telomerase RNA component, telomere

## Abstract

Telomerase, which maintains the ends of chromosomes, consists of two core components, the telomerase reverse transcriptase (*TERT*) and the telomerase RNA (*TERC*). Haploinsufficiency for *TERC* or *TERT* leads to progressive telomere shortening and autosomal dominant dyskeratosis congenita (DC). The clinical manifestations of autosomal dominant DC are thought to occur when telomeres become critically short, but the rate of telomere shortening in this condition is unknown. Here, we investigated the consequences of *de novo TERT* gene deletions in a large cohort of individuals with 5p– syndrome. The study group included 41 individuals in which the chromosome deletion resulted in loss of one copy of the *TERT* gene at 5p15.33. Telomere length in peripheral blood cells from these individuals, although within the normal range, was on average shorter than in normal controls. The shortening was more significant in older individuals suggesting an accelerated age-dependent shortening. In contrast, individuals with autosomal dominant DC due to an inherited *TERC* gene deletion had very short telomeres, and the telomeres were equally short regardless of the age. Although some individuals with 5p– syndrome showed clinical features that were reminiscent of autosomal dominant DC, these features did not correlate with telomere length, suggesting that these were not caused by critically short telomeres. We conclude that a *TERT* gene deletion leads to slightly shorter telomeres within one generation. However, our results suggest that several generations of *TERT* haploinsufficiency are needed to produce the very short telomeres seen in patients with DC.

## Introduction

Telomerase is a ribonucleoprotein complex that maintains the ends of chromosomes. It consists of two core components, the telomerase reverse transcriptase (*TERT*) and the telomerase RNA (*TERC*), that serves as a template for the synthesis of telomeric DNA repeats at chromosome ends (Greider & Blackburn, 1987). Loss of telomerase activity leads to progressive telomere shortening and loss of telomere integrity (Blackburn, 2001). When telomeres become critically short, cell cycle arrest or cell death occurs (Kim *et al*., 1994). Haploinsufficiency for *TERC* causes the autosomal dominant variant of dyskeratosis congenita (DC) (Vulliamy *et al*., 2001).

DC is a rare inherited bone marrow failure syndrome. In addition to progressive bone marrow failure, clinical features include nail dystrophy, abnormal skin pigmentation and mucosal leukoplakia (Knight *et al*., 1998). Patients with DC have very short telomeres (Vulliamy *et al*., 2001), suggesting that excessive premature telomere shortening is responsible for the development of the disease in these individuals (for review, see Mason *et al*., 2005). More recently, *TERT* gene mutations have been identified in rare individuals with autosomal dominant DC and in sporadic cases of bone marrow failure (Armanios *et al*., 2005; Vulliamy *et al*., 2005; Yamaguchi *et al*., 2005; Liang *et al*., 2006; Vulliamy & Dokal, 2006; Xin *et al*., 2007). In autosomal dominant DC, the age of onset is earlier and the disease is more severe in later generations who carry the pathogenic mutation, a phenomenon known as anticipation (Vulliamy *et al*., 2001; Mason, 2003). Disease anticipation in autosomal dominant DC is thought to be due to the inheritance of increasingly short telomeres in subsequent generations (Vulliamy *et al*., 2004; Goldman *et al*., 2005). The rate of telomere shortening has been extensively studied in telomerase-deficient mice. Mice with no telomerase function, either because of a *Terc* gene deletion (*Terc* RNA–/– mice) or due to a null mutation in the *Tert* gene (*Tert–*/– mice), showed no obvious phenotype in the early generations of inbreeding despite progressive telomere shortening. Three to six generations of inbreeding were necessary to produce telomeres sufficiently short to cause a ‘short telomere phenotype’ (Blasco *et al*., 1997; Herrera *et al*., 1999; Liu *et al*., 2000; Niida *et al*., 2000). The rate of telomere shortening in human haploinsufficiency for *TERC* or *TERT* is not known. Similarly, the number of generations that of haploinsufficiency and increasingly short telomeres has to be inherited before telomeres become critically short is unknown.

To explore the impact of *de novo TERT* haploinsufficiency on telomere shortening, we studied a large number of individuals with 5p– syndrome having a *TERT* gene deletion. The 5p– syndrome, also know as Cri du Chat syndrome, or monosomy 5p, is one of the most frequent autosomal deletion syndromes. The deletions involve the short arm of chromosome 5 and usually encompass *TERT* gene located at 5p15.33 (Meyerson *et al*., 1997; Zhang *et al*., 2003). This syndrome has several phenotypic components including the characteristic cry that gives the syndrome its name, facial dysmorphology, speech delay and mental retardation (Mainardi *et al*., 2006). Clinical manifestations vary with the size and location of the chromosomal deletion (Overhauser *et al*., 1994; Gersh *et al*., 1995; Kim & Wu, 1997; Mainardi *et al*., 2006). The deletion in individuals with 5p– syndrome is either a *de novo* event or is inherited from a parent with a balanced translocation (Mainardi *et al*., 2006). Consequently, individuals with 5p– syndrome whose deletion includes the *TERT* gene are the first generation to be haploinsufficient for *TERT* (*de novo TERT* gene deletion). Classical features of DC have not been associated with the syndrome; however, only few individuals have been followed into adulthood and no hematological assessments have been performed.

We were, therefore, interested in the degree of telomere shortening in individuals with 5p– syndrome due to the phenomenon of anticipation in autosomal dominant DC and in whether haploinsufficiency for *TERT* may contribute to the clinical spectrum of the 5p– syndrome. Our investigations demonstrate that the majority of individuals with 5p– had only one copy of the *TERT* gene. Telomeres measured in peripheral blood cells from individuals with 5p– syndrome and a *TERT* gene deletion, although significantly shorter than age-matched controls, remained within normal levels and did not approach the severely shortened telomeres seen in patients with autosomal dominant DC. We conclude that a *de novo TERT* gene deletion, although associated with shorter telomeres, does not lead to critically short telomeres within one generation and that clinical manifestations of the 5p– syndrome are unlikely to be caused by premature shortening of telomeres.

## Results

### Individuals with 5p– syndrome have clinical manifestations consistent with premature aging

Fifty-one families with at least one family member affected with the 5p– syndrome participated in our study. Fifty-two participants had 5p– syndrome; 79 were unaffected family members. The median age of participants with 5p– syndrome was 9 years old (range, 1–42 years), and that of unaffected family members (parents and siblings) was 41 years old (range, 2–70 years). All individuals with 5p– syndrome showed typical clinical features (Mainardi *et al*., 2006) including, on review, the cat-like cry and specific alteration of the voice, facial anomalies (i.e. epicanthal folds, microretrognathia), and psychomotor and mental retardation. In addition to the classical features of 5p– syndrome, ridging of the fingernails was noted in 14 individuals with 5p– syndrome and early hair graying in 10 individuals. Both ridging of the fingernails and early hair graying are early cutaneous manifestations characteristic of DC. The youngest participant with ridged fingernails was 2 years old and the youngest with early graying of the hair was 12 years old. Telomere lengths in individuals with 5p– syndrome having ridged finger/toe nails were not significantly different from the individuals without ridged nails (*P* = 0.92). Similarly, there was no statistically significant difference of telomere length between individuals with 5p– syndrome having early hair graying/loss and without this feature (*P* = 0.80). Leukoplakia, another characteristic manifestation of DC, was not seen in any of the participants with 5p– syndrome.

### Deletion of one copy of *TERT* in individuals with 5p– syndrome

**Fig. 1:**
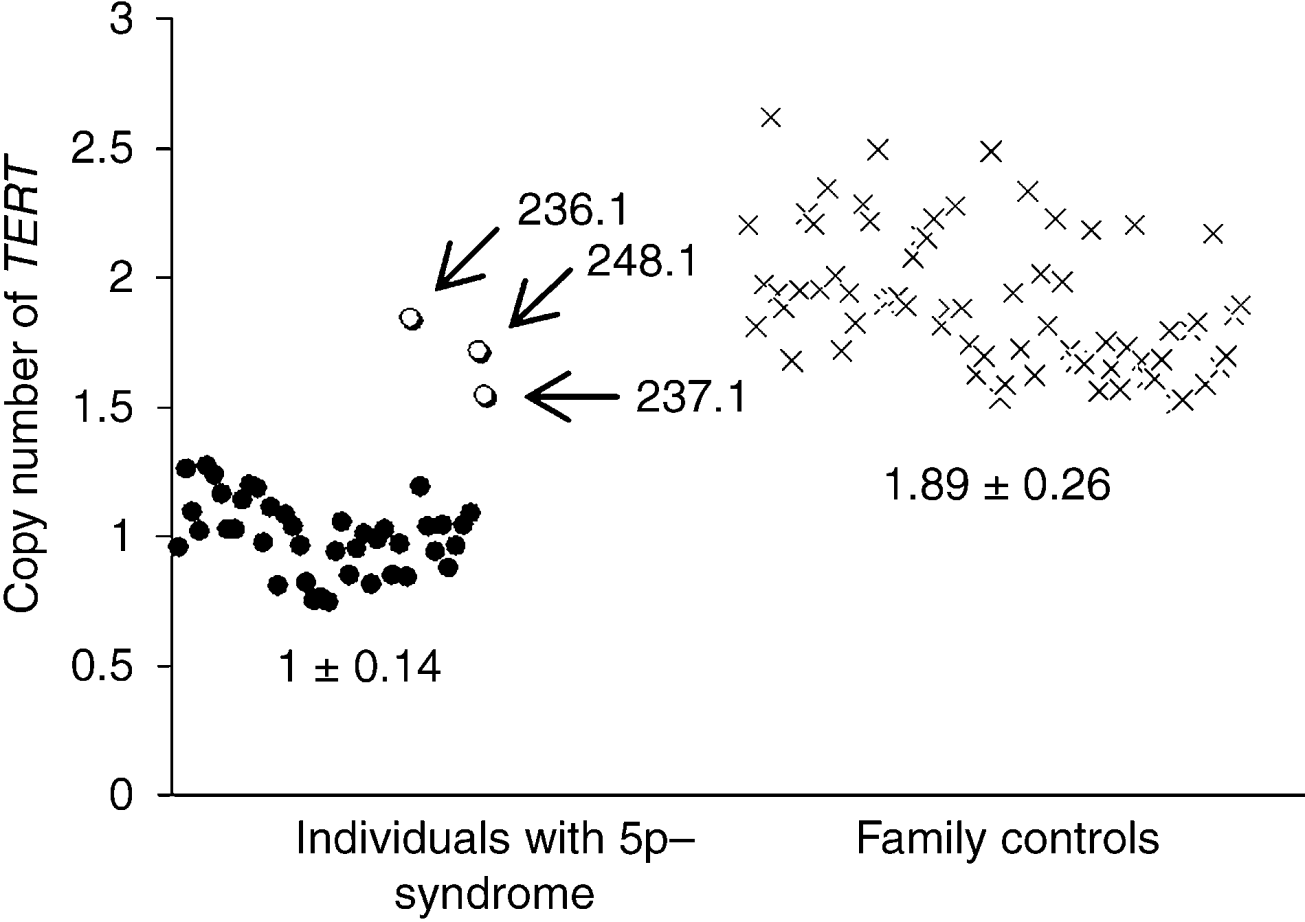
Copy number of *TERT* in individuals with 5p– syndrome and family members. Gene copy number was determined by quantitative polymerase chain reaction (PCR). The majority of 5p– individuals have only one copy of the *TERT* gene (•, *n* = 41), whereas there are two copies of *TERT* in family members (×, *n* = 70). In three individuals with 5p– syndrome (○, *n* = 3), two copies of *TERT* were identified, suggesting that 5p– was due to interstitial deletion on 5p–.

**Fig. 2:**
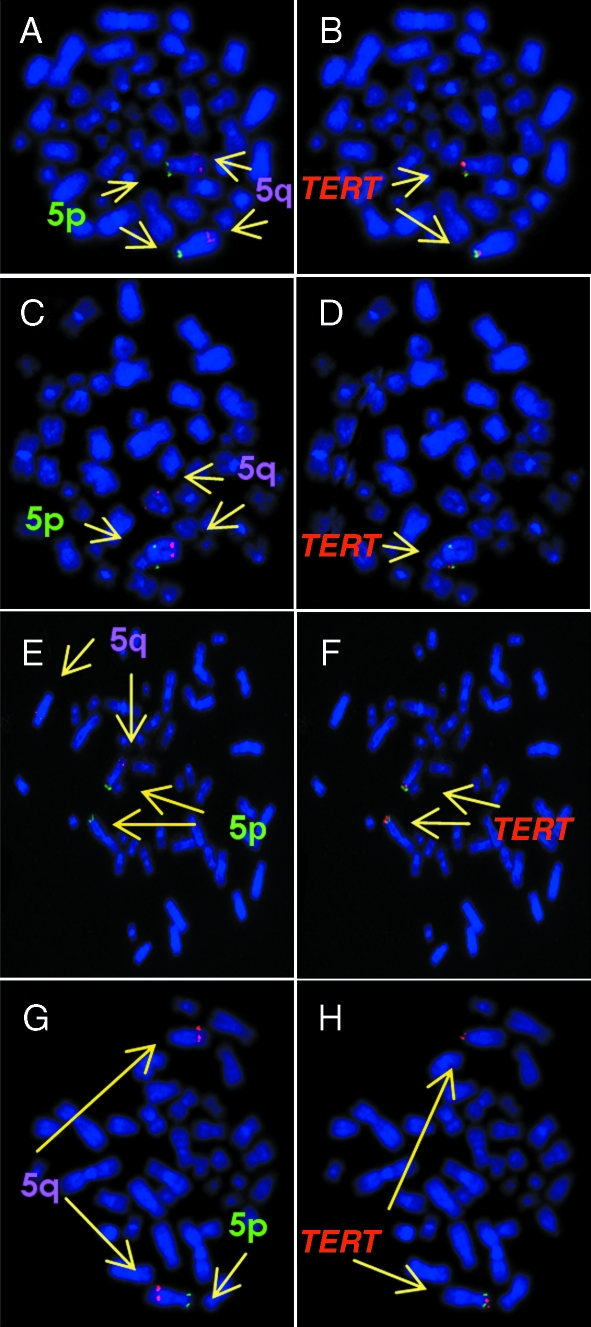
Fluorescent *in situ* hybridization (FISH) of metaphase chromosomes from individuals with 5p– syndrome and their family members. Representative FISH analysis of lymphocyte metaphase from a normal control (A, B), an individual with 5p– syndrome and a *TERT* deletion (C, D), a parent with balanced translocation (E, F), and an individual with 5p– syndrome having an interstitial deletion but two copies of *TERT* (G, H) are shown. The *TERT*, 5p15.2 and 5q31 markers were labeled by red, green and pink, respectively. Two copies of 5p (A, B), 5q (A) and the *TERT* gene (B) are found in the normal individual. Metaphase chromosomes from the individual with 5p– syndrome and with a terminal deletion include one copy of 5p and one copy of *TERT* (C, D). A parent of an individual with 5p syndrome shows a balanced translocation of 5p, with one of the 5p and 5q not on the same chromosome (E); two copies of *TERT* are present (F). Representative example of an individual with an interstitial deletion with only one copy of 5p but two copies of the TERT is shown in (G, H).

We determined the copy number of the *TERT* gene by quantitative polymerase chain reaction (Q-PCR). While the 79 unaffected family members had an average copy number of *TERT* of 1.89 ± 0.26, the 42 individuals with 5p– syndrome had an average of 1.00 ± 0.14 (*P* = 4.5 × 10^−39^, Fig. 1). The result suggests that there is a concomitant deletion of *TERT* in the majority of participants having deletion of 5p. We identified three individuals with 5p– having two copies of the *TERT* gene (Fig. 1, open circles), indicating an interstitial deletion sparing the *TERT* locus was responsible for the syndrome, as confirmed by fluorescence *in situ* hybridization (FISH) analysis. Representative examples are shown in Fig. 2. Figure 2A,B shows FISH analysis of activated lymphocytes from an unaffected family member with two copies of 5p and two copies of 5q, labeled by green and pink, respectively. The two copies of the *TERT* gene hybridizing to the respective probe are shown in red (Fig. 2B). Figure 2C,D shows an example of an individual with 5p– whose deletion of chromosome 5p includes the *TERT* gene. Figure 2E,F illustrates an example of a balanced translocation including chromosome 5p in an unaffected parent who has a child with 5p– due to the inheritance of an unbalanced translocation. In the parent, 5p and 5q are found on two different chromosomes, similarly, the *TERT* gene is found on two different chromosomes. Figure 2G,H shows FISH analysis of metaphases of lymphocytes from an individual with 5p– due to an interstitial deletion with two hybridization signals for the *TERT* gene, but on one chromosome 5 the hybridization signal for 5p is missing.

In humans after birth *TERT* has been shown to be expressed in germ cells, stem cells and their immediate progeny, and in activated lymphocytes and monocytes (Kim *et al*., 1994; Feng *et al*., 1995; Nakamura *et al*., 1997; Masutomi *et al*., 2003). To investigate whether haploinsufficiency for the *TERT* gene is associated with a reduced level of *TERT* mRNA and reduced levels of telomerase activity, we measured the levels of *TERT* mRNA and telomerase activity in activated T cells after incubations with anti-CD3 and anti-CD28 antibodies. The levels of *TERT* mRNA as measured by Q-PCR and of telomerase activity as determined by Q-PCR (Kim & Wu, 1997; Uehara, 2006) were highly variable and not significantly different in individuals with 5p– syndrome vs. normal controls (see Supplementary Fig. S1A). However, our finding of accelerated telomere shortening (see below) indirectly indicates that functional haploinsufficiency is likely to operate in individuals with 5p– syndrome.

### Shortened telomeres in individuals with 5p– syndrome

**Fig. 3:**
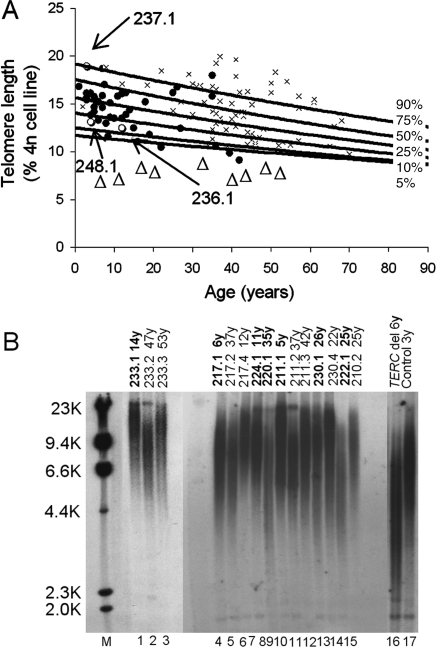
Telomere lengths in individuals with 5p– syndrome and normal study controls. (A) Telomere length of peripheral blood mononuclear (PBMN) cells from individuals with 5p– syndrome (•, *n* = 44), family members (×, *n* = 69), and dyskeratosis congenita patients carrying a *TERC*-3′ deletion (▴, *n* = 9) were measured by flow cytometry fluorescence *in situ* hybridization (FISH). The lines represent the 90th, 75th, 50th, 25th, 10th and 5th percentile of the telomere length in healthy controls (*n* = 105). The three 5p– individuals with two copies of *TERT* are labeled (○). Telomere length was expressed as the mean fluorescence of PBMN relative to a tetraploid cell line. (B) Telomere length in peripheral blood cells from individuals with 5p– syndrome and family members (parents and siblings) determined by in-gel hybridization. The first number indicates the family number; the second number indicates the family relation to the proband. Individuals with 5p– syndrome are shown in bold and were assigned. .1, .2 represents the mother; .3 the father; and .4 the sibling within one family. The age of each individual is shown. Lanes 16 and 17 show the telomere length of a DC patient with a *TERC* gene deletion, and one of his unaffected family members without the deletion. Note the very short telomeres in the DC patient with a *TERC* deletion (Lane 16) as compared to the telomere lengths seen in 5p– individuals.

It has been well established that telomere length shortens with age in somatic cells (Harley *et al*., 1990; Hastie *et al*., 1990; Nordfjall *et al*., 2005). Telomere lengths were determined from 105 healthy individuals aged from 3 to 94 years. The 5th, 10th, 25th, 50th, 75th and 90th percentiles of the age-dependent distribution of telomere lengths are shown in Fig. 3. We have used the general linear model (GLM) procedure to analyze the association between telomere length and *TERT* gene deletion, age, gender and race. Consistent with previous reports, we found a negative association between telomere lengths and age (*P* < 0.001). In contrast, gender and race were not significant predictors of telomere lengths (*P* = 0.993 and 0.239, respectively). Although at first glance telomere lengths in individuals with 5p– seemed not to differ much from normal controls by either flow cytometry FISH or Southern blot analysis (Fig. 3A,B), statistical analysis revealed a significant association between telomere length and the copy number of *TERT* (*P* = 0.0066). Furthermore, age-dependent telomere shortening in individuals with 5p– syndrome and a concomitant *TERT* gene deletion seemed to be accelerated compared to normal controls (*r* = –0.09, *P* = 0.0046 vs. *r* = –0.07, *P* < 0.001), but this did not reach statistical significance due to the low number of older individuals with 5p– syndrome and the wide distribution of telomere lengths [95% confidence interval (CI): –0.15 to –0.03 vs. –0.09 to –0.05]. However, when compared to telomere lengths from individuals with autosomal dominant DC due to a *TERC* gene deletion, whose telomere lengths are far below the 5th percentile (see Fig. 3A,B), telomere lengths in blood cells from individuals with 5p– syndrome and a *TERT* gene deletion were only marginally shorter than normal controls (see Fig. 3A,B). We have also quantitatively estimated the differences in the rate of telomere shortening. It was found that telomere lengths were shortened by 1.1 relative fluorescence units (RFU) in the first generation of individuals with 5p– syndrome compared to normal controls (*P* = 0.0147). In contrast, in the autosomal dominant DC patients with a *TERC* gene deletion telomere lengths were on average 5.6 RFU lower than in individuals with 5p– syndrome (*P* < 0.0001).

### Normal peripheral blood cell counts but a decreased number of circulating hematopoietic progenitor cells in individuals with 5p– syndrome

**Fig. 4:**
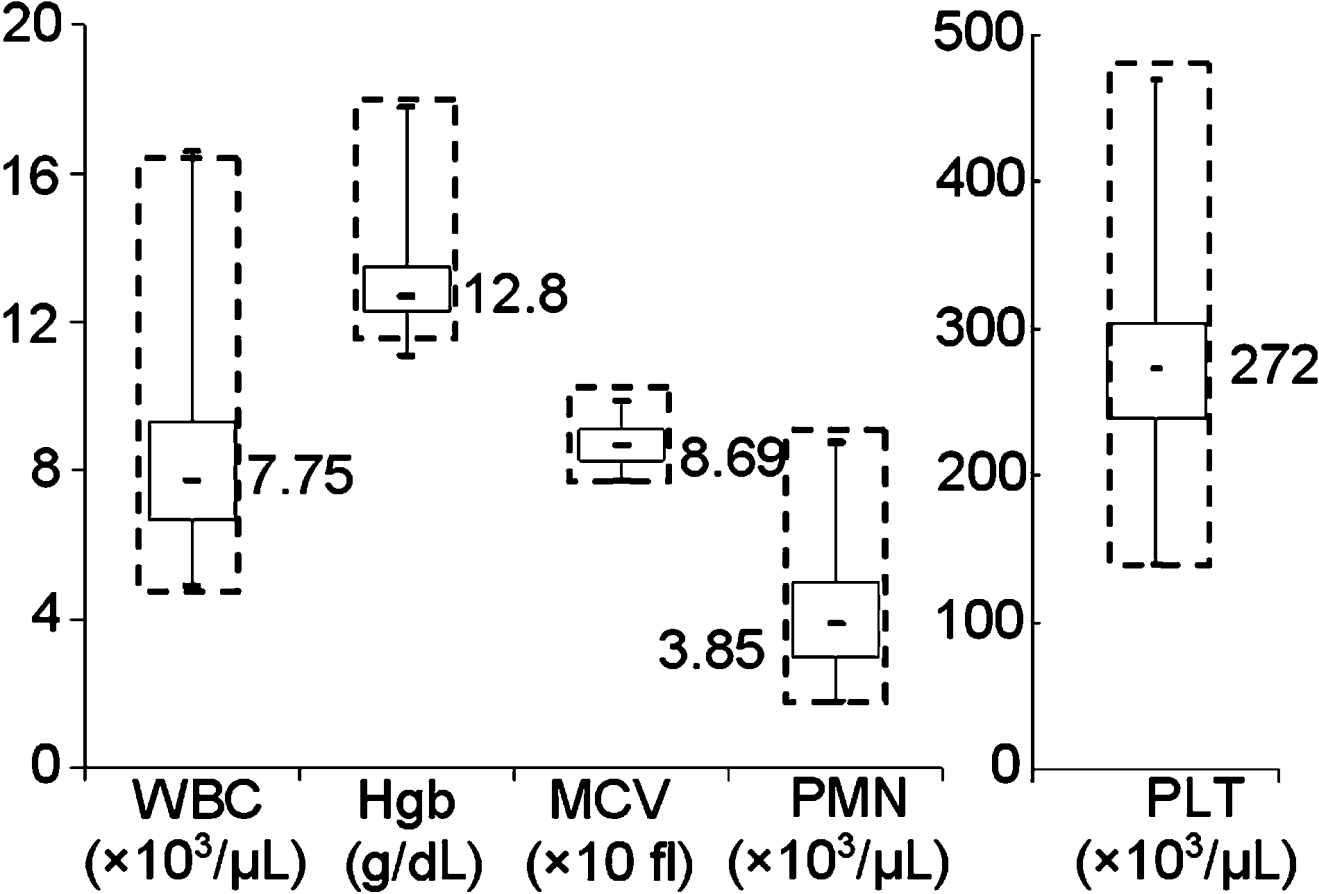
Blood values from individuals with 5p– syndrome. Blood values obtained from 40 individuals with 5p– syndrome are shown. Each box plot represents the minimum, the 25% percentile, the median, the 75% percentile and the maximum of blood values. Median values are indicated. WBC, white blood cell counts; Hgb, hemoglobin; PMN, polymorphonuclear cells; PLT, platelets; MCV, mean corpuscular volume of red cells. Boxes with broken line represent normal range.

The majority of individuals with DC develop signs of bone marrow failure (Marrone *et al*., 2005; Mason *et al*., 2005), which is thought to be due to a deficiency of telomerase in hematopoietic stem cells and their immediate progeny. We therefore wanted to know whether a *TERT* gene deletion in individuals with 5p– syndrome was associated with impaired hematopoiesis. Bone marrow biopsy was deemed inappropriate on ethical grounds, so we used peripheral blood as a surrogate to evaluate the effect of the *TERT* gene deletion on hematopoiesis (see Fig. 4). With the exception of four individuals who had slightly decreased hemoglobin levels (11–13 g dL^−1^ respectively), peripheral blood values were normal for hemoglobin (13.07 ± 1.28 g dL^−1^), white blood cell counts (8.33 ± 2.7 × 10^3^ per µL), platelet count (277.44 ± 72.82 × 10^3^ per µL), mean corpuscle volume (87.23 ± 5.05 fl), and polymorphonuclear cells count (4.07 ± 1.59 × 10^3^ per µL).

**Fig. 5:**
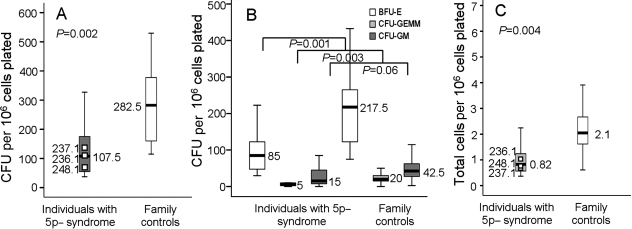
Circulating progenitor cells in individuals with 5p– syndrome and study controls. Each box plot represents the minimum, the 25% percentile, the median, the 75% percentile and the maximum. Median values are indicated. (A) The number of colony forming units (CFU) formed from peripheral blood was less in 5p– individuals compared to family members (*n* = 17, *P* = 0.0012). (B) Among the three types of colonies identified: burst forming unit-erythroid (BFU-E), colony forming unit–granulocyte, macrophage (CFU-GM), and CFU–granulocyte, erythrocyte, monocyte, megakaryocyte (CFU-GMMM), a significant decrease in 5p– individuals was found in BFU-Es (*P* = 0.001) and CFU-GEMMs (*P* = 0.033). (C) Total number of cells in cell culture is significantly decreased in 5p– individuals compared to family members (*P* = 0.004).

As an additional parameter we also analyzed the number of circulating hematopoietic progenitor cells in this study, using a clonogenic progenitor assay. Surprisingly, the number of colonies formed in individuals with 5p– syndrome was significantly less than in unaffected family members (Fig. 5A; median 21.5 vs. 56.5; *P* = 0.0012). Among the three colony types identified, burst-forming units–erythroid (BFU-E) and colony forming units–granulocyte, erythrocyte, monocyte, megakaryocyte (CFU-GEMM) showed a significant decrease in individuals with 5p– syndrome (Fig. 5B;*P* = 0.001 and 0.033, respectively), whereas the reduction in colony-forming unit–granulocyte, macrophage (CFU-GM) did not reach statistical significance when compared to family members (*P* = 0.06). The size of the colonies formed by progenitors from individuals with 5p– syndrome were similar to those formed by normal controls as demonstrated by a proportional decrease of cell numbers harvested from the respective culture plates (Fig. 4C;*P* = 0.004). There was no significant correlation between telomere length and the number of circulating progenitor cells (*r* = 0.306, *P* = 0.094).

## Discussion

There is an ongoing debate about the extent to which *TERT* and *TERC* are limiting for telomere length (Liu *et al*., 2000; Chiang *et al*., 2004; Hao *et al*., 2005a). Here we investigated the effect of a *TERT* gene deletion on telomere lengths in individuals with 5p– syndrome. Our studies showed that telomere lengths in individuals with 5p– syndrome, who are haploinsufficient for the *TERT* gene, are only slightly shorter than telomere lengths in normal controls, who have two copies of *TERT*. Shorter telomeres were previously demonstrated by Zhang and colleagues in eight individuals with 5p– syndrome compared to eight normal controls, with the conclusion that significant telomere shortening was evident in 5p– and might contribute to the 5p– phenotype (2003). However, the study did not take into account the wide distribution of telomere lengths in the normal population and lacks sufficient numbers of normal controls and 5p– individuals to evaluate the extent of telomere shortening in these individuals. In our study, telomere lengths of 44 individuals with 5p– syndrome were compared with a much larger group of control individuals (*n* = 105). Both populations showed a wide distribution of telomere lengths, which were largely overlapping. These results, in contrast to the previous study, put the telomere shortening observed in individuals with 5p– into proportion, indicating that telomere shortening in individuals with 5p– syndrome, although significant, is minimal and that the majority of telomere lengths of individuals with 5p– syndrome still fall within the distribution of normal telomere lengths (5th–95th percentile, see Fig. 3A). Comparing telomere lengths of individuals with 5p– syndrome to the telomere lengths in individuals with autosomal dominant DC due to a *TERC* gene deletion, we find that individuals with 5p– have much longer telomeres than individuals with autosomal dominant DC and a *TERC* gene deletion, both by flow cytometry FISH and by Southern blotting (see Fig. 3A,B).

The most likely explanation for these findings is that in individuals with 5p– syndrome, haploinsufficiency for *TERT* almost always occurs *de novo* (Mainardi *et al*., 2006) and, as demonstrated here, within one generation this does not lead to the excessively short telomeres seen in individuals with autosomal dominant DC due to an inherited *TERC* gene deletion. These findings strongly support the hypothesis of anticipation and underline the importance of anticipation in the pathogenesis of disease in patients with autosomal dominant DC.

Anticipation due to the inheritance of increasingly shorter telomeres is a unique characteristic of autosomal dominant DC. It explains the increasing severity and younger age of onset of the disease in later generations in patients with autosomal dominant DC (Vulliamy *et al*., 2001, 2004; Mason, 2003; Armanios *et al*., 2005; Goldman *et al*., 2005). The cumulative effect of telomere shortening over several generations also explains why even a minimal impairment of telomerase activity will eventually lead to telomeres sufficiently short to cause disease.

An alternative explanation of our finding could be that haploinsufficiency for *TERC* has a different effect on telomere length from haploinsufficiency for *TERT*. In mice, *TERT* and *Terc* RNA deficiency showed a very similar rate of telomere shortening and an almost identical phenotype after several generations of inbreeding (Blasco *et al*., 1997; Liu *et al*., 2000). However, telomere dynamics differ in mouse and humans. In contrast to humans, mice *Terc* RNA or *Tert* haploinsufficiency is not associated with the development of a disease phenotype (Blasco *et al*., 1997; Liu *et al*., 2000; Chiang *et al*., 2004; Hathcock *et al*., 2005), whereas in humans homozygosity or compound heterozygosity for telomerase null mutations have never been described and are most likely not compatible with life. We can not exclude that *TERC* and *TERT* haploinsufficiency might have a different effect on telomere lengths in humans. Samples from patients with autosomal dominant DC due to a *TERT* gene mutation were not available to us; but the fact that autosomal dominant DC occurs in families with inherited *TERC* or *TERT* gene mutations strongly supports our hypothesis that in the majority of humans neither haploinsufficiency for *TERT* nor for *TERC* leads to excessively short telomeres or autosomal dominant DC disease in the first generation.

We expected that telomere shortening due to *TERT* haploinsufficiency would be most significant during embryonic development, but we were surprised to find that telomere lengths in children with 5p– syndrome below the age of 5 years were not significantly different to normal controls (see Fig. 3), but telomere loss appeared to be accelerated thereafter. This raises the interesting question of when in human life telomerase haploinsufficiency affects telomere length the most. Interestingly, individuals with autosomal dominant DC due to *TERC* gene deletions have very short telomeres already at a very young age (see Fig. 3A). We hypothesize that there is a lower limit of telomere length for a functional blood cell and that lower limit is reached in autosomal dominant DC patients with disease (Goldman *et al*., 2005). Blood progenitor cells with shorter telomeres stop dividing or die leading to the bone marrow failure in individuals with autosomal dominant DC.

Interestingly, some individuals with 5p– showed ridged fingernails and early graying of their hair, a clinical manifestation associated with premature aging. Similarly, individuals with 5p– syndrome had low numbers of circulating peripheral blood progenitor cells compared to normal controls, which is suggestive for bone marrow failure. However, fingernail changes were also found in one of the three individuals with 5p– syndrome having two copies of the *TERT* gene, and equally low numbers of circulating blood progenitor cells were present in all three 5p– individuals with two copies of *TERT*. Early graying of hair was not seen in individuals with 5p– syndrome and with two copies of *TERT,* but all three were below the age of 10. Although we cannot formally exclude that some of these features are caused by short telomeres in the specific tissue, the fact that some of these features were found also in individuals with 5p– but two copies of *TERT* suggests that the observed changes of the nails, early hair graying, and the decreased number of circulating hematopoietic progenitor cells are due to haploinsufficiency of one or more of the other genes involved in the large chromosomal deletion. Similarly, although none of the affected genes other than *TERT* is involved in telomere maintenance, we cannot exclude that their haploinsufficiency directly or indirectly influences telomere length.

In summary, our data demonstrate accelerated telomere shortening in individuals with 5p– syndrome. The residual telomere length, rather than reduced telomerase activity, has been implicated to be responsible for the phenotype in telomerase deficient mice (Hao *et al*., 2005b). Short telomeres have also been implicated in the pathology of patients with DC. Our analysis demonstrates that within one generation, telomeres do not become sufficiently short to cause disease or contribute to the 5p– phenotype. However, whether TERT haploinsuffciency contributes to the 5p– phenotype outside of its role on telomeres remains to be determined (Blackburn, 2005).

## Experimental procedures

### Human subjects

The study group comprised 52 individuals with 5p– syndrome from 51 families: 37 individuals (36 families) were enrolled during the annual meeting of the 5p– Society held in St. Louis, MO, USA, in July 2005, and 15 individuals (15 families) were enrolled during the annual meeting held in Santa Clara, CA, USA, in 2006. Seventy-nine family members (parents and siblings) were included as ‘intrastudy controls’. All individuals with 5p– syndrome had been previously diagnosed by cytogenetic analysis in clinically approved laboratories. The diagnosis of 5p– syndrome was confirmed by review of medical records. In selected cases the diagnosis was additionally confirmed by FISH using the commercially available LSI D5S23, D5S721/5q31 LSI EGR1 probe (Vysis XY, Downers Grove, IL, USA; see also below). After informed consent was obtained, individuals were examined with a particular emphasis on the presence of classical features associated with DC (abnormal skin pigmentation, dystrophic fingernails, leukoplakia and early graying of the hair), and blood was drawn from a peripheral vein. Forty-five blood samples were collected from the participants with 5p– syndrome and 79 from the study controls. Blood samples were analyzed within 36 h. Approval for the study was obtained by the Washington University School of Medicine Institutional Review Board.

### Q-PCR for detection of a *TERT* gene deletion

Genomic DNA was extracted from peripheral blood leukocytes with the DNA blood mini kit (Qiagen, Valencia, CA, USA). Aliquots of 20 ng DNA were assayed with a SYBR Green core reagent kit (Applied Biosystems, Foster City, CA, USA). The primer sequences for the *TERT* gene were: F: 5’-ACGAGCACCGTCTGATTAGG-3’; R: 5’-GGGTTCTTCCAAACTTGCTG-3’. β-globin gene served as the reference gene in the determination of the copy number of *TERT* (Cawthon, 2002). All PCRs were performed on the Prism 7000 Sequence Detection System (Applied Biosystems) following the instructions of the manufacturer. Serial dilutions (5–80 ng) of reference DNA (a pool of DNA samples) were used to plot the standard curve for the *TERT* gene and reference gene β-G. The 2^−ΔΔC^_T_ method was adopted to analyze the data (Livak & Schmittgen, 2001). Individual copy number of *TERT* was expressed as the ratio of *TERT/β-G* of each sample relative to the average ratio of individuals with 5p– syndrome.

### Telomere length measurement by flow cytometric FISH

Telomere length of peripheral blood mononuclear (PBMN) cells isolated by Ficoll–Hypaque gradient centrifugation was measured as previously described (Goldman *et al*., 2005) using fluorescin (FITC)-conjugated (C_3_TA_2_)_3_ peptide nucleic acid (PNA, Applied Biosystems) probe with a flow cytometer (Beckman Coulter, Fullerton, CA, USA). Relative telomere length was determined by comparing isolated PBMC with a control cell line (GM03671C; Coriell Institute of Medical Research, Camden, NJ, USA), a tetraploid cell line, which served as an internal control and was assigned a telomere length of 100%. Telomere lengths of 105 healthy individuals (ranging from 3 to 94 years old) were used to determine the distribution of age-dependent telomere length.

### Terminal restriction fragment length analysis by in-gel hybridization

The average telomere restriction fragment length of genomic DNA was determined as previously described (Goldman *et al*., 2005). Briefly, 3 µg of genomic DNA digested with restriction enzymes *Rsa* I and *Hinf* I (Invitrogen, Carlsbad, CA, USA) were electrophoresed on a 0.5%/0.6 × TBE agarose gel along with Lambda *Hind* III marker. After gel drying, denaturing and neutralization, gels were hybridized to a γ^32^P-end-labeled telomeric probe. The average telomere restriction fragment length was determined using the Imagequant™ software.

### FISH analyses

The diagnosis of 5p– syndrome and the deletion of the *TERT* gene were confirmed by FISH analysis of metaphase chromosomes prepared from fresh PBMC or Epstein-Barr virus-transformed B lymphocytes as previously described with minor modifications (Goldman *et al*., 2005). Metaphase spreads were subjected to the hybridization procedure using a Biotin-labeled *TERT* probe synthesized from a previously isolated and entirely sequenced BAC spanning the whole *TERT* locus (Leem *et al*., 2002), by a nick translation kit (Vysis). The slides were then incubated with avidin-Texas red (Vector laboratories, Burlingame, CA, USA) followed by antiavidin antibody (Vector laboratories) to amplify the signal. After double staining with a commercially available LSI D5S23, D5S721/5q31 LSI EGR1 probe (Vysis), the metaphase spreads were counterstained with 4′,6-diamidino-2-phenylindole (DAPI) and visualized under a fluorescence microscope (Nikon, Melville, NY, USA).

### Measurement of telomerase activity in CD3/CD28-stimulated peripheral blood T cells

PBMN cells (10^6^) were mixed with 25 µL Dynabeads CD3/CD28 T cell expander (Invitrogen) and further stimulated with 50 U mL^−1^ rIL-2 (Pharmingen, Franklin Lakes, NJ, USA). The stimulated cells were removed from the beads using a magnet (Invitrogen) and stored at –80 °C until use. Protein was extracted from stimulated cells by CHAPS XL lysis buffer (Chemicon, Temecula, CA, USA) and quantified using the RC DC Protein assay kit (Bio-Rad, Hercules, CA, USA). Telomerase activity was measured by a Q-PCR based telomeric repeat amplification protocol (TRAP) assay (Kim & Wu, 1997; Uehara, 2006) with minor modifications. Thermal cycling reactions were performed with the Prism 7000 Sequence Detection System (Applied Biosystems).

### *TERT* mRNA levels in CD3/CD28-stimulated T cells from peripheral blood

RNA was extracted from CD3/CD28-stimulated T cells by the RNeasy mini kit (Qiagen). cDNA was synthesized from 2 µg RNA using Superscript III RT (Invitrogen). cDNA samples were subjected to Q-PCR using a SYBR Green core reagent kit (Applied Biosystems) along with the primers: TERT-F: 5’-GCCGATTGTGAACATGGAC; TERT-R: GCTGAACAGTGCCTTCACC. In parallel, each sample was run with primers: G3PDH-F: 5’-GAAGGTGAAGGTCGGAGTC; G3PDH-R; 5’-GAAGATGGTGATGGGATTTC as internal control. The amount of *TERT* mRNA was quantified by the 2^−ΔΔC^_T_ method as described above.

### Progenitor assay

PBMN cells (2 × 10^5^) were plated in duplicates in 1 mL methylcellulose medium (MethoCult GF H4434, Stem Cells Technologies, Tukwila, WA, USA) and incubated at 37 °C. Colonies were scored and counted after 10 days of culture. The cells were subsequently collected and counted using a Coulter counter (Beckman Coulter).

### Statistical analysis

All analyses were performed using the SAS computer software (version 9.1, SAS Institute, Cary, NC, USA). Generalized linear model (SAS GLM procedure) was used to assess whether *TERT* deletion was a significant predictor of telomere length, adjusted for the effects of age, gender and race. The telomere lengths among groups (subjects with 5p– syndrome, unaffected family controls and DC patients carrying one copy deletion of *TERC*) were compared by using Duncan's and Tukey's tests. Furthermore, nonindependence of family members was adjusted by using the robust covariance matrix estimator, which asymptotically yielded the same parameter estimates, but the standard errors and associated test were corrected for the dependence. The method assumed same degree of dependency among all members of each family. The Kruskal–Wallis nonparametric test followed by Dunnett C test was used for the comparison of CFU between 5p– individuals and family controls. In this report, *P* < 0.05 was considered statistically significant.

## Supplementary material

The following supplementary material is available for this article:

Fig. S1 (A) *TERT* RNA expression level in activated T lymphocytes from individuals with 5p- syndrome and family controls. The RNA level of *TERT* was determined by quantitative polymerase chain reaction (Q-PCR) analysis and expressed as the ratio relative to the RNA level of *GAPDH* gene. There was no statistically significant difference between the individuals with 5p- syndrome (•, median: 0.00026; *n* = 12) and the family controls (×, median: 0.00026; *n* = 14) (*P* = 0.91). Individuals with 5p- but two copy numbers of *TERT* are shown. The horizontal line indicates the median. (B) Telomerase activity in activated T lymphocytes from individuals with 5p- syndrome and family controls. Telomerase activity was determined by Q-PCR analysis. TPG (total products generated) is defined as the amount of template [TSR7: AATCCGTCGAGCAGAGTTAG(GGTTAG)6] that is extended with telomeric repeats by telomerase. There is no statistically significant difference between the individuals with 5p- syndrome (•, median: 0.00099; *n* = 14) and the family controls (×, median: 0.0018; *n* = 16) (*P* = 0.46). Individuals with 5p- but two copy numbers of *TERT* are shown. The horizontal line indicates the median.

This material is available as part of the online article from: http://www.blackwell-synergy.com/doi/abs/10.1111/j.1474-9726.2007.00324.x

(This link will take you to the article abstract).

## References

[b1] Armanios M, Chen JL, Chang YP, Brodsky RA, Hawkins A, Griffin CA, Eshleman JR, Cohen AR, Chakravarti A, Hamosh A, Greider CW (2005). Haploinsufficiency of telomerase reverse transcriptase leads to anticipation in autosomal dominant dyskeratosis congenita. Proc. Natl Acad. Sci. USA.

[b2] Blackburn EH (2001). Switching and signaling at the telomere. Cell.

[b3] Blackburn EH (2005). Telomeres and telomerase: their mechanisms of action and the effects of altering their functions. FEBS Lett.

[b4] Blasco MA, Lee HW, Hande MP, Samper E, Lansdorp PM, DePinho RA, Greider CW (1997). Telomere shortening and tumor formation by mouse cells lacking telomerase RNA. Cell.

[b5] Cawthon RM (2002). Telomere measurement by quantitative PCR. Nucleic Acids Res.

[b6] Chiang YJ, Hemann MT, Hathcock KS, Tessarollo L, Feigenbaum L, Hahn WC, Hodes RJ (2004). Expression of telomerase RNA template, but not telomerase reverse transcriptase, is limiting for telomere length maintenance *in vivo*. Mol. Cell. Biol.

[b7] Feng J, Funk WD, Wang SS, Weinrich SL, Avilion AA, Chiu CP, Adams RR, Chang E, Allsopp RC, Yu J, Le S, West MD, Harley CB, Andrews WH, Greider CN, Villeponteau B (1995). The RNA component of human telomerase. Science.

[b8] Gersh M, Goodart SA, Pasztor LM, Harris DJ, Weiss L, Overhauser J (1995). Evidence for a distinct region causing a cat-like cry in patients with 5p deletions. Am. J. Hum. Genet.

[b9] Goldman F, Bouarich R, Kulkarni S, Freeman S, Du HY, Harrington L, Mason PJ, Londono-Vallejo A, Bessler M (2005). The effect of TERC haploinsufficiency on the inheritance of telomere length. Proc. Natl Acad. Sci. USA.

[b10] Greider CW, Blackburn EH (1987). The telomere terminal transferase of Tetrahymena is a ribonucleoprotein enzyme with two kinds of primer specificity. Cell.

[b11] Hao LY, Armanios M, Strong MA, Karim B, Feldser DM, Huso D, Greider CW (2005a). Short telomeres, even in the presence of telomerase, limit tissue renewal capacity. Cell.

[b12] Hao ZM, Luo JY, Cheng J, Li L, He D, Wang QY, Yang GX (2005b). Intensive inhibition of hTERT expression by a ribozyme induces rapid apoptosis of cancer cells through a telomere length-independent pathway. Cancer Biol. Ther.

[b13] Harley CB, Futcher AB, Greider CW (1990). Telomeres shorten during ageing of human fibroblasts. Nature.

[b14] Hastie ND, Dempster M, Dunlop MG, Thompson AM, Green DK, Allshire RC (1990). Telomere reduction in human colorectal carcinoma and with ageing. Nature.

[b15] Hathcock KS, Jeffrey Chiang Y, Hodes RJ (2005). *In vivo* regulation of telomerase activity and telomere length. Immunol. Rev.

[b16] Herrera E, Samper E, Martin-Caballero J, Flores JM, Lee HW, Blasco MA (1999). Disease states associated with telomerase deficiency appear earlier in mice with short telomeres. EMBO J.

[b17] Kim NW, Piatyszek MA, Prowse KR, Harley CB, West MD, Ho PL, Coviello GM, Wright WE, Weinrich SL, Shay JW (1994). Specific association of human telomerase activity with immortal cells and cancer. Science.

[b18] Kim NW, Wu F (1997). Advances in quantification and characterization of telomerase activity by the telomeric repeat amplification protocol (TRAP). Nucleic Acids Res.

[b19] Knight S, Vulliamy T, Copplestone A, Gluckman E, Mason P, Dokal I (1998). Dyskeratosis congenita (DC) registry: identification of new features of DC. Br. J. Haematol.

[b20] Leem SH, Londono-Vallejo JA, Kim JH, Bui H, Tubacher E, Solomon G, Park JE, Horikawa I, Kouprina N, Barrett JC, Larionov V (2002). The human telomerase gene: complete genomic sequence and analysis of tandem repeat polymorphisms in intronic regions. Oncogene.

[b21] Liang J, Yagasaki H, Kamachi Y, Hama A, Matsumoto K, Kato K, Kudo K, Kojima S (2006). Mutations in telomerase catalytic protein in Japanese children with aplastic anemia. Haematologica.

[b22] Liu Y, Snow BE, Hande MP, Yeung D, Erdmann NJ, Wakeham A, Itie A, Siderovski DP, Lansdorp PM, Robinson MO, Harrington L (2000). The telomerase reverse transcriptase is limiting and necessary for telomerase function *in vivo*. Curr. Biol.

[b23] Livak KJ, Schmittgen TD (2001). Analysis of relative gene expression data using real-time quantitative PCR and the 2(–ΔΔ C(T)) method. Methods.

[b24] Mainardi PC, Pastore G, Castronovo C, Godi M, Guala A, Tamiazzo S, Provera S, Pierluigi M, Bricarelli FD (2006). The natural history of Cri du Chat syndrome. A report from the Italian Register. Eur. J. Med. Genet.

[b25] Marrone A, Walne A, Dokal I (2005). Dyskeratosis congenita: telomerase, telomeres and anticipation. Curr. Opin. Genet. Dev.

[b26] Mason PJ (2003). Stem cells, telomerase and dyskeratosis congenita. Bioessays.

[b27] Mason PJ, Wilson DB, Bessler M (2005). Dyskeratosis congenita – a disease of dysfunctional telomere maintenance. Curr. Mol. Med.

[b28] Masutomi K, Yu EY, Khurts S, Ben-Porath I, Currier JL, Metz GB, Brooks MW, Kaneko S, Murakami S, DeCaprio JA, Weinberg RA, Stewart SA, Hahn WC (2003). Telomerase maintains telomere structure in normal human cells. Cell.

[b29] Meyerson M, Counter CM, Eaton EN, Ellisen LW, Steiner P, Caddle SD, Ziaugra L, Beijersbergen RL, Davidoff MJ, Liu Q, Bacchetti S, Haber DA, Weinberg RA (1997). hEST2, the putative human telomerase catalytic subunit gene, is up-regulated in tumor cells and during immortalization. Cell.

[b42] Mosse YP, Greshock J, Margolin A, Naylor T, Cole K, Khazi D, Hii G, Winter C, Shahzad S, Asziz MU, Biegel JA, Weber BL, Maris JM (2005). High-resolution detection and mapping of genomic DNA alterations in neuroblastoma. Genes Chromosomes Cancer.

[b30] Nakamura TM, Morin GB, Chapman KB, Weinrich SL, Andrews WH, Lingner J, Harley CB, Cech TR (1997). Telomerase catalytic subunit homologs from fission yeast and human. Science.

[b31] Niida H, Shinkai Y, Hande MP, Matsumoto T, Takehara S, Tachibana M, Oshimura M, Lansdorp PM, Furuichi Y (2000). Telomere maintenance in telomerase-deficient mouse embryonic stem cells: characterization of an amplified telomeric DNA. Mol. Cell. Biol.

[b32] Nordfjall K, Larefalk A, Lindgren P, Holmberg D, Roos G (2005). Telomere length and heredity: Indications of paternal inheritance. Proc. Natl Acad. Sci. USA.

[b33] Overhauser J, Huang X, Gersh M, Wilson W, McMahon J, Bengtsson U, Rojas K, Meyer M, Wasmuth JJ (1994). Molecular and phenotypic mapping of the short arm of chromosome 5: sublocalization of the critical region for the cri–du–chat syndrome. Hum. Mol. Genet.

[b34] Uehara H (2006). Real-time detection and quantification of telomerase activity utilizing energy transfer primers. Methods Mol. Biol.

[b35] Vulliamy T, Dokal I (2006). Dyskeratosis congenita. Semin. Hematol.

[b36] Vulliamy T, Marrone A, Goldman F, Dearlove A, Bessler M, Mason PJ, Dokal I (2001). The RNA component of telomerase is mutated in autosomal dominant dyskeratosis congenita. Nature.

[b37] Vulliamy T, Marrone A, Szydlo R, Walne A, Mason PJ, Dokal I (2004). Disease anticipation is associated with progressive telomere shortening in families with dyskeratosis congenita due to mutations in TERC. Nat. Genet.

[b38] Vulliamy TJ, Walne A, Baskaradas A, Mason PJ, Marrone A, Dokal I (2005). Mutations in the reverse transcriptase component of telomerase (TERT) in patients with bone marrow failure. Blood Cells Mol. Dis.

[b39] Xin ZT, Beauchamp AD, Calado RT, Bradford JW, Regal JA, Shenoy A, Liang Y, Lansdorp PM, Young NS, Ly H (2007). Functional characterization of natural telomerase mutations found in patients with hematological disorders. Blood.

[b40] Yamaguchi H, Calado RT, Ly H, Kajigaya S, Baerlocher GM, Chanock SJ, Lansdorp PM, Young NS (2005). Mutations in TERT, the gene for telomerase reverse transcriptase, in aplastic anemia. N. Engl. J. Med.

[b41] Zhang A, Zheng C, Hou M, Lindvall C, Li KJ, Erlandsson F, Bjorkholm M, Gruber A, Blennow E, Xu D (2003). Deletion of the telomerase reverse transcriptase gene and haploinsufficiency of telomere maintenance in Cri du chat syndrome. Am. J. Hum. Genet.

