# Supplementary figures and images for: Telomerase reverse transcriptase haploinsufficiency and telomere length in individuals with 5p– syndrome
Source: Aging Cell. 2007 Oct 1;6(5):689–97. doi: 10.1111/j.1474-9726.2007.00324.x (PMC2583393; doi:10.1111/j.1474-9726.2007.00324.x)

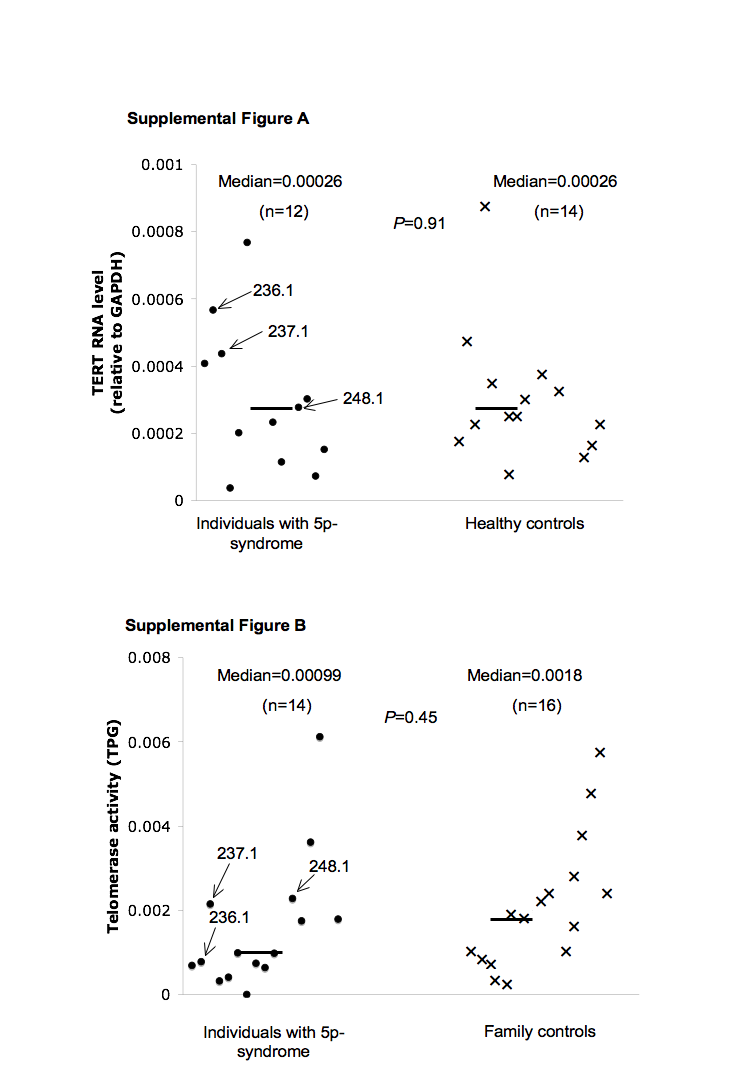

Supplement: Fig. S1 — (A) TERT RNA expression level in activated T lymphocytes from individuals with 5p- syndrome and family controls. The RNA level of TERT was determined by quantitative polymerase chain reaction (Q-PCR) analysis and expressed as the ratio relative to the RNA level of GAPDH gene. There was no statistically significant difference between the individuals with 5p- syndrome (•, median: 0.00026; n = 12) and the family controls (×, median: 0.00026; n = 14) (P = 0.91). Individuals with 5p- but two copy numbers of TERT are shown. The horizontal line indicates the median. (B) Telomerase activity in activated T lymphocytes from individuals with 5p- syndrome and family controls. Telomerase activity was determined by Q-PCR analysis. TPG (total products generated) is defined as the amount of template [TSR7: AATCCGTCGAGCAGAGTTAG(GGTTAG)6] that is extended with telomeric repeats by telomerase. There is no statistically significant difference between the individuals with 5p- syndrome (•, median: 0.00099; n = 14) and the family controls (×, median: 0.0018; n = 16) (P = 0.46). Individuals with 5p- but two copy numbers of TERT are shown. The horizontal line indicates the median. [file ace0006-0689-SupFigAB.tif]
